# The Effects of COVID-19 Pandemic on Stress Vulnerability of Nursing Students According to Labour Market Status

**DOI:** 10.3390/healthcare9060633

**Published:** 2021-05-27

**Authors:** Mihaela Simionescu, Angelo Pellegrini, Elena-Nicoleta Bordea

**Affiliations:** 1 Institute for Economic Forecasting, Romanian Academy, 050711 Bucharest, Romania; 2 Faculty of Midwifery and Nursing, “Carol Davila” University of Medicine and Pharmacy, 020021 Bucharest, Romania; pellegrini.angelo@gmail.com (A.P.); lilianabordea2015@gmail.com (E.-N.B.)

**Keywords:** occupational stress, COVID-19, pandemic, employment, nursing students

## Abstract

Nursing has always been a stressful job affecting the physical and mental health of the nurses, but the new medical context of the COVID-19 pandemic has enhanced this issue. The objective of this paper is to study differences between non-employed and employed nursing students in the medical system of Romania before and during the epidemic using matching methods and a sample of 526 nursing students. Stressors and strategies for coping with stress were also identified during the pandemic. The results indicated that employed students were more vulnerable to stress compared to non-employed ones before and during the pandemic. Females registered significantly higher stress scores compared to males in the period of the epidemic. The analysis of the overall sample suggested that gender and environment were significant stress factors during the pandemic, but living conditions were not relevant. The most effective coping strategies against stress for nurses during the COVID-19 crisis were based on self-control and the spiritual dimension, unlike the previous period when other colleagues’ support helped most nurses to overcome difficult working conditions. The implications of this study might help medical management in ensuring a less stressful environment for nurses during the epidemic.

## 1. Introduction

The COVID-19 pandemic represents a major threat for public health and nurses who are key actors in the prevention, treatment and control of the coronavirus. Uncertainty related to the SARS-CoV-2 epidemic has enhanced the levels of stress, depression and anxiety in the general population, including nurses [1]. According to an online survey in 20 countries, adults were more affected by stress during the COVID-19 pandemic, rather than depression and anxiety [2]. Medical personnel need good mental health in the fight against various infectious diseases [3]. Stress reduces immunity, making nurses more vulnerable to the coronavirus. Most studies concerning the effects of the COVID-19 pandemic are related to epidemiological factors, but only a few papers have focused on the stress that affects nurses [4,5,6,7,8,9].

Nursing is considered among the most stressful jobs in the world, the negative effects referring not only to the physical and spiritual health of the individuals, but also unfavorable consequences on the medical system, including patients [10]. Positive beliefs of individuals related to the future evolution of the pandemic are associated to post-traumatic growth, while negative beliefs are connected to post-traumatic symptoms [11].

A classification of 130 professions that could generate mental health issues made by the U.S. Occupational Safety and Health Institute placed nursing at 27th place, with a higher degree of stress than other categories of medical personnel [12]. Previous studies confirmed an average or high level of stress for nurses in India (87.4% individuals in a sample), Iran (57.4%) and Saudi Arabia (45.5%) [13].

Job stress consists of emotional and physical effects related to the contact of the labor force with the working environment when there is a significant disparity between demands related to the job and the capacity to accomplish these requests [14]. Actually, the stress appears when there is a higher volume of job demands compared to the person’s various resources, from a physical, emotional, social, psychological or economic point of view [15]. The consequences of stress among the nurses were mentioned by Moustaka and Constantinidis: anxiety, weak motivation for work, worse decision-making, apathy, decrease in quality of life, risk of chronic diseases, and less immunity [16].

Job stress is specific to modern society having negative consequences from a medical, spiritual, social and economic point of view. The stress affects people’s bodies and souls, as well as social interactions, reducing labor productivity [17].

Given these negative effects, studies analyzing the stress among nurses are focused on a few directions: factors that determine stress and strategies to cope with stress. Therefore, our paper will focus on the identification of factors that determine stress among nursing students, and differences between employed and non-employed individuals in the sample before and during the pandemic. Moreover, a comparison between coping strategies for stress before and during the pandemic for nurses in the sample is made. The novelty of this research with respect to previous achievements from the literature: The comparison between nurses and non-employed nursing students in terms of vulnerability to stress, the comparative analysis before and during the pandemic for these categories, the differences between coping strategies for stress before the epidemic and during it. Moreover, the novelty is also supported by methodology compared to previous studies that are based on the multiple regression model [5,9]. In our case, matching methods were used to ensure a deep analysis of the differences in the vulnerability to stress among different categories of nursing students, some of them already employed as nurses due to their secondary education.

Various determinants of stress for nurses have been identified in the period before the COVID-19 pandemic: institutional pressure [14], scenes of violence in their relationship with patients and night shift work [15], patients with serious diseases and emergency cases (Rahimi et al., 2004), and dissensions with colleagues and a weak quality of teamwork [14]. Three quarters of nurses working in the Zagazig Fever Hospital in Egypt experienced a high level of stress at the beginning of the COVID-19 pandemic. In this particular case, the identified stressors refer to: potential death, fears and personal demands, stigma, and the use of biosecurity measures [18].

For the period corresponding to the COVID-19 pandemic, several studies based on surveys identified stressors for nurses. A few studies focused on demographic characteristics, which is also an objective of this paper. For example, in Turkey, the analysis of a sample of 662 nursing students during the epidemic indicated that stress is determined by age, stress, but also to specific issues related to the COVID-19 pandemic [4]. The age was considered a good predictor for stress among nurses from Jordan during the pandemic in the sense that younger ones are more vulnerable to stress [6]. Gender was a predictor of stress in the case of nurses from India, females being two times more vulnerable to stress than males [19]. A similar study was conducted in this paper, but additional research was done for employed and non-employed nursing students, the employed ones already working as nurses in medical units. Other studies focused on personal states of nurses and labor requirements. An example is the study of Mo et al. for nurses in China where anxiety, the number of children in the family, and working hours per week are significant stressors [5]. Wang et al. showed that sources of stress during the coronavirus epidemic included the fear of being infected and transmitting the virus to one’s family, and also the discomfort of wearing a mask all the time [8]. The most important sources of stress for nurses in China mentioned by Leng et al. are: fear of being infected with the coronavirus, issues related to protective equipment, hard work, lack of experience in the new context, and the isolated environment [20]. Homesickness was found to be the most important stressor for nurses in a hospital from Shanghai [9]. Personal traits might also act as stressors for all people, including nurses. Liu et al. analyzed the connection between perceived stress and personality features in the context of the COVID-19 pandemic [21]. Canadian adults with extroversion and neuroticism were more vulnerable to stress during the epidemic compared to other categories, but also compared to the period before the pandemic.

Given the negative consequences of work stress for nurses, different recommendations for coping with stress were made in various studies. Before the pandemic, Murat et al. showed that control over the issues that might determine stress levels was a better solution than a defensive strategy [22]. Other recommendations were made in the case of a current medical crisis. Altruism and devotion were found to be the main pillars that help Chinese nurses to cope with stress during the pandemic (Wang et al., 2020). Coping self-efficacy helped nurses from Jordan to reduce stress during the SARS-CoV-2 crisis [6]. Pasay-An identified a few strategies to reduce stress among nurses in Saudi Arabia: support from managers and colleagues and good communication of new procedures during the pandemic [23]. However, Folkman and Moskowitz explained that all these quantitative approaches should be completed by a qualitative analysis to figure out individual options related to coping with stress [24].

The aim of this study is to compare the vulnerability to stress between employed and non-employed nursing students from Romania using a total sample of 526 students before and during the COVID-19 pandemic. Some stress factors and practical strategies to cope with stress are also identified based on the individuals’ responses. The conceptual framework is based on a sample of nursing students that was analyzed before the COVID-19 pandemic and a sample studied during the epidemic. The analysis is focused on a few characteristics (gender, environment, and status on the labor market) that serve in identifying some stressors for these students. Moreover, some strategies to cope with stress are indicated for employed nursing students during the epidemic.

## 2. Methodology

In Romania, students can be hired in the medical sector as nurses with high school and post-high-school education in this field (sanitary school). Some of them might choose higher education in this domain due to a better salary and the opportunity to get a position in the management of a medical unit. Some students in the sample worked in the medical sector, while other ones did not have a job. There were a few nursing students that were employed in another domain, but they did not make the subject of our analysis.

The objective of this survey was to assess nursing students’ vulnerability according to various characteristics in the pre-pandemic and pandemic period. The survey base was given by the register of student enrolments. The observation unit of the population is represented by students. The sample unit is nursing students from Bucharest. Ethical principles were respected, such as informed consent (the participants were informed about the scope of the research, duration, content, and voluntary implication in responding to the questions of the questionnaire), confidentiality (the participants cannot be identified), and anonymity (participants’ names are not presented). There are no potential threats to internal and external validity. Construct validity is ensured, since the measures have been validated in previous studies. The Miller and Smith scale was previously used in measuring vulnerability to stress. Missing data were not registered for the analyzed sample.

The research is based on an online questionnaire in Romanian language that was distributed to the nursing students through a private link. The questionnaire consisted of 26 questions related to items used to assess vulnerability to stress, and the rest of the questions refer to demographic characteristics. This study focused on undergraduate nursing students from the Faculty of Midwifery and Nursing located in Bucharest, Romania in the “Carol Davila” University of Medicine and Pharmacy. The analysis was conducted in two different periods: before the COVID-19 pandemic (May–June 2019) and during the epidemic (December 2020) on two samples of nursing students (sample before the pandemic and sample during the epidemic). Some of the students were included in both samples.

The population in this research is the undergraduate nursing students (Bachelor students) from Bucharest in the single faculty with this specialization in this city. The volume of the population in the academic year 2018/2019 was 360 students in May–June 2019, but we selected only the nursing students in their second, third and fourth academic year. The reason for considering only the students in these years is related to the fact that there were more students who were employed compared to those in the first year. Only 263 of the nursing students in their second, third and fourth year out of 270 were non-employed or worked as nurses, and they agreed to answer this questionnaire. Students who worked in fields other than the medical sector were not included in our sample.

From a statistical point of view, we have a survey based on volunteering. During the COVID-19 pandemic, the same number of undergraduate students was selected out of 270 students in their second, third and fourth year, and they fulfilled the criteria of working as nurses or being non-employed. A limitation of this technique is the lack of representativeness of the sample at a national level, but the analysis is relevant for nursing students from Bucharest.

A few hypotheses were tested on these samples in order to identify differences between employed and non-employed students, females and males, and individuals belonging to rural or urban environments in what concerned stress vulnerability before and during the pandemic.

The stress scores were computed using the Miller and Smith scale for responses to 20 questions in the questionnaire. These questions referred to biological characteristics (health status perception, weight), psychological characteristics (type of personality), and behavioural, economic and social factors (lifestyle, social support, income, religion, coffee drink, smoking, and excessive alcohol consumption) [25]. For each question, the response should be associated with one of the five categories: never (5 points), rarely (4 points), sometimes (3 points), often (2 points), and almost always (1 point). The Alpha (Cronbach) coefficient is used to measure the internal consistency between the items of the scale. The value of the Cronbach’s Alpha coefficient is 0.78, which indicates good consistency.

Potential sources of bias are given by the lack of honesty in answering some questions related to the capacity to efficiently organize the time or regular affection received from other people.

The volume of the total population is 360 (*N*). The sample size (*n*) is determined based on a qualitative variable (gender). Since the population is not homogenous, the mean of the qualitative characteristic in population (*p*) is 0.5. The maximum permissible error limit ($\Delta_{W}$) is ±5%. The Z score is considered for a 5% level of significance (*z* = 1.96).

$$n=\frac{z^{2}\cdot p\left(1-p\right)}{\Delta_{w}^{2}+\frac{z^{2}\cdot p\left(1-p\right)}{N}}=\frac{1.96^{2}\cdot 0.25}{0.05^{2}+\frac{1.96^{2}\cdot 0.25}{360}}=\frac{0.9604}{0.0009+0.0026}=$$

$$=\frac{0.9604}{0.0035}≅274\;\mathrm{students}$$

Each sample had a lower volume (263) since we do not have more students to fulfil all the criteria of eligibility in their second, third and fourth year. The sample representativeness is checked for this sample using a *z* test.

The share of females in the sample (*w*) is 78%, while the share of females in the population (*p*) is 74%.

The hypotheses of the *z* test are:

H_0_: *w* = *p*

H_1_: w≠p

The computed value of *z* is:

$$z_{calculated}=\frac{w-p}{\sqrt{\frac{p\cdot \left(1-p\right)}{n}}}=\frac{0.78-0.74}{\sqrt{\frac{0.1716}{263}}}=\frac{0.04}{0.0255}=1.565$$

Since $\left|z_{calculated}\right|<1.96$, the null hypothesis is not rejected at the 5% level of significance. Therefore, our sample is representative of the population of nursing students from Bucharest.

The following categories of vulnerabilities to stress were considered based on the computed scores:

- 0–10 points—resistance to stress;
- 11–29 points—weak vulnerability to stress;
- 30–49 points—medium vulnerability to stress;
- 50–74 points—high vulnerability to stress;
- 75–80 points—extreme vulnerability to stress.

There are other questions used to identify the demographic characteristics:

- Age group (18–25 years, 26–29 years, 30–39 years, 40–49 years, 50–65 years);
- Gender (female/male);
- Marital status (married/unmarried);
- Status on labor market (employed/non-employed);
- Environment (urban/rural environment);
- Living conditions (alone, on rent, with parents, in student house).

In this study, D represents a dummy variable that helps us to identify the treated and control group. More dummy variables are constructed to check more hypotheses: status on the labor market (1 for employed nursing students, 0 for non-employed nursing students), COVID-19 pandemic (1 for students that took the survey during the pandemic, 0 for students that took the survey before the COVID-19 epidemic), gender (1 for male, 0 for female), and environment (1 where the student belongs to an urban environment, 0 where the student belongs to a rural environment).

More hypotheses were considered, and various matching methods were applied to validate or invalidate these assumptions. Sensitivity analyses were not considered.

**Hypothesis** **1** **(H1).** *Employed nursing students are more vulnerable to stress compared to non-employed ones during the COVID-19 pandemic*.

**Hypothesis** **2** **(H2).** *Employed nursing students during the COVID-19 pandemic are more vulnerable to stress compared to employed ones before this epidemic*.

**Hypothesis** **3** **(H3).** *Employed females present a higher level of stress compared to employed males before and during the COVID-19 pandemic*.

**Hypothesis** **4** **(H4).** *Non-employed females present a higher level of stress compared to non-employed males before and during the COVID-19 pandemic*.

**Hypothesis** **5** **(H5).** *Employed females present a higher level of stress compared to non-employed females before and during the COVID-19 pandemic*.

**Hypothesis** **6** **(H6).** *Employed males present a higher level of stress compared to non-employed males before and during the COVID-19 pandemic*.

**Hypothesis** **7** **(H7).** *Females were more stressed than males during the pandemic*.

**Hypothesis** **8** **(H8).** *The vulnerability to stress is influenced by living conditions or environment during epidemic*.

The average stress score for the entire sample is 25.63, which suggests low vulnerability to stress. However, during the pandemic, the mean of stress scores was 27.55 with values ranging from 6 to 58. Of the nursing students analyzed during the pandemic, 41.03% had average or high vulnerability to stress, which supports the research of this sample in order to provide suitable recommendations for coping with stress in this context. According to Table 1, most of the nursing students are females (78.21%). More than half of the respondents lived in cities, and almost three quarters of them are between 18 and 25 years old. Of the individuals, 88.33% were unmarried and more than half lived with their parents. Only 35.9% of them had a job.

## 3. Results

The effects of the students’ status on stress scores and the labor market were evaluated for the entire sample covering the period before the epidemic and during the pandemic, but also in each of these periods.

In Table 2, the impact of three dummy variables on vulnerability to stress is shown: status on labor market (employed/non-employed), gender (male/female) and environment (urban/rural environment). The control variables for explaining stress based on employment are represented by gender, environment, marital status, and age group. For comparisons between students by gender, the control variables refer to age category, marital status, and environment, while the control variables for students according to environment are age group, marital status, and gender.

In the sample during the pandemic, employed nursing students presented higher stress scores by an average of almost 6 points from the average of 26.79 points for unemployed students. The employed nursing students during the SARS-CoV-2 epidemic had higher stress scores by an average of almost 5 points from the mean of 26.17 points that corresponded to non-employed students. Females were more vulnerable to stress compared to males during the pandemic by an average of almost 5 points from the average level of 24.03 for men. Students from rural environments presented higher stress scores by around 5 points from the average of 25.14 points for those living in a city.

All in all, a few conclusions could be drawn from the results above:

- During the pandemic, the nursing students with a job were significantly more vulnerable to stress compared to the non-employed ones with a tendency of medium vulnerability to stress for employed individuals and a low vulnerability to stress for non-employed ones;
- The nursing students employed during the COVID-19 epidemic were significantly more vulnerable to stress (medium vulnerability to stress) compared to those that worked before the pandemic (weak vulnerability to stress);
- Females were significantly more vulnerable to stress than males;
- Students belonging to a rural environment were more vulnerable to stress (medium vulnerability) than those coming from cities (low vulnerability).

According to propensity-score-matching in Table 3, there are no significant differences between employed females and non-employed females before the COVID-19 pandemic according to stress vulnerability. The same result was obtained for males. One possible explanation for this situation is the fact that both males and females were more stressed during the pandemic regardless of their status in the labor market.

According to Table 4, there are no significant differences between employed females and employed males before the pandemic and non-employed females and non-employed males before the COVID-19 pandemic. Therefore, we can state that there are no differences between females and males according to their status in the labor market before the epidemic. However, this situation has changed during the pandemic. Non-employed females are more vulnerable to stress compared to non-employed males, while employed females have greater stress scores compared to employed males.

In the sample during the pandemic, employed females presented higher stress scores by an average of around 5 points from the average of 23.77 points for employed males. The non-employed females during the SARS-CoV-2 epidemic had higher stress scores by an average of almost 5 points from the mean of 24.03 points that correspond to non-employed males.

All in all, a few conclusions are stated based on Table 4:

- During the pandemic, the employed females were significantly more vulnerable to stress compared to the employed males, while the non-employed females were more affected by stress than non-employed males;
- There were no significant differences in terms of vulnerability to stress between employed males and females, respectively, and non-employed males and females before the COVID-19 pandemic.

The ANOVA procedure has been applied to check for significant factors affecting the stress scores during the epidemic. More factors were considered: environmental and living conditions as Table 5 shows. The significant influence of the environment on stress has already been confirmed by the previous analysis.

According to the ANOVA analysis, only gender and environment had a significant influence on stress scores, but living conditions did not have a significant influence on vulnerability to stress during COVID-19 at a 5% level of significance. These results might be explained by the fact that gender and environment are more relevant for students, rather than living conditions.

Starting from the initial hypotheses, the following results were obtained:

- *Employed nursing students are more vulnerable to stress compared to non-employed ones during the COVID-19 pandemic*.
- *Employed nursing students during the COVID-19 pandemic are more vulnerable to stress compared to employed ones before this epidemic*.
- *Employed females present a higher level of stress compared to employed males during the COVID-19 pandemic, but not before epidemic*.
- *Non-employed females present a higher level of stress compared to non-employed males during the COVID-19 pandemic, but not before epidemic*.
- *Employed females did not present a higher level of stress compared to non-employed females before and during the COVID-19 pandemic*.
- *Employed males did not present a higher level of stress compared to non-employed males before and during the COVID-19 pandemic*.
- *Females were more stressed than males during the pandemic*.
- *The vulnerability to stress is influenced by environment, but not by living conditions during epidemic*.

A few strategies to cope with stress were proposed to employed nursing students before and during the pandemic, such as medical management, spirituality, self-control, seeking help from colleagues, and family support. Before the COVID-19 pandemic, 46.67% of the nurses benefited from colleagues’ support to cope with stress, 16.67% of them had family support, 13.33% considered spirituality as vital to overcome difficult situations at work, 2% of them considered self-control as the most important strategy, and only 0.33% relied on medical management. During the pandemic, the hierarchy has changed: self-control (40%), spirituality (23.33%), family support (16.67%), seeking help from colleagues (10%), and medical management (10%). It is clear that nurses did not have confidence in medical management in both periods, and improvements are necessary in this field. If self-control did not have a significant importance in coping with stress before the pandemic, during this period the nurses had to adapt and have internal motivation to fight with stressful situations. On the other hand, spirituality gained a higher position, while requests for colleagues’ support significantly reduced.

## 4. Discussion

The discussion will be focused on comparisons with previous studies regarding stressors and methods to cope with stress, but also on new findings that are different from other previous approaches.

Gender was identified as an important stress factor during the medical crisis, as in other studies by Aslan and Pekince [4] for Turkey and Wilson et al. [19] for India. Moreover, in our research we identified a quantitative measure for this difference between males and females using matching scores. In this case, the stress scores for women were, on average, by 5 points more than those of men. Our findings are similar to previous studies that showed the higher vulnerability of females to stress compared to males [26]. Results by gender should be treated with caution. There is a large difference between men and women, typical of the profession.

Unlike other studies, the environment was identified as a stress factor for Romanian nursing students during the pandemic. This result is confirmed by expectations, since people living in rural environments in Romania are more likely to be affected by poverty and social exclusion than those in cities. The isolation brought by the epidemic and new challenges from those commuting from a village to a city for work purposes in this period represented additional sources of stress.

Strategies used to cope with stress during the pandemic are similar with those proposed by Akbar et al. [12] and Skinner and Zimmer-Gembeck [27] for the period before the medical crisis, and those of Shahrour and Dardas [6] for the crisis period. Skinner and Zimmer-Gembeck promoted more self-regulation as an essential part of self-control [27]. Akbar et al. explained that the control strategy was related to nurses’ good health which improves immunity, making them less vulnerable to any virus [12]. Self-efficiency was promoted by in these difficult times for medical systems [6]. Our findings are contrary to those of Pasay-An for the pandemic period [23], since medical management and colleagues’ support were less appreciated coping strategies for Romanian nurses in the sample. Therefore, we can observe that in our cases, nurses focused more on internal than external resources to surpass the difficult working conditions.

The control methods that could be efficient for nurses refer to toleration, exercise, positive thinking, self-reliance, recreation, self-learning, compassion for those patients in worse health states, staying far from additional stressful situations, and prayer (13, 28]. All these methods are designed to alleviate the mental, physical, and psychological comfort of nurses.

Besides self-control, spirituality was also found to play a major role in the nurses’ initiatives to cope with job stress. This strategy supposes more levels according to Akbar et al.: an individual approach based on personal opinions, primary and secondary stages (recognition of God’s power), valuable resources (for example, time spent in nature), religious behavior (making prayers and reading holy books), and meaning-making (spiritual rebuilding) [12]. The importance of the spiritual dimension in the management of stress in the case of nurses was highlighted by Jannati et al. for clinical nurses [28], by Harris for nurses working in hospices [29], and Bakibinga et al. for nurses in general [30].

According to the analysis of Elloker, it seems that institutional, co-worker, and family support are less efficient in coping with stress, even if authorities and families play a central role [31]. Therefore, nursing managers should make more effort to identify and solve the issues of nurses and ensure there is emotional support, as Shirey recommended [32]. Colleagues should also be more open to supporting nurses in this difficult situation, and this could be achieved by institutional initiatives, as Chang et al. suggested [3]. Besides the higher chances of getting infected with the new coronavirus, family should ensure the emotional support of relatives who are nurses.

Besides these recommendations, robust policies are required to ensure a suitable physical and structural environment. Medical management should follow a few targets for nurses: interactive management, flexible scheduling, job security, harmonious labor relations, and efficient communication practices [33]. For their implementation, these policies need investments in order to prevent diseases and maintain good health for nurses. Educational initiatives are also welcome in order to provide information about maintaining good health, strategies to cope with stress, and a good work–life balance.

Specific strategies are also necessary for those nursing students that are not already employed, but are preparing to enter the labor market. Therefore, they need a strong psychological preparation to face the stressful situations at work. The lack of experience will be for sure an important stressor, but working in the pandemic will be an additional challenge. Educational initiatives and the share of experience of professors and employed colleagues could help them to cope with future job stress. These students should be motivated to work in the medical system since Romania has a significant deficit in their labor force in this domain because of emigration in more developed countries that offer better salaries. From this point of view, faculties should adapt their educational programs in real-time to include topics about coping with stress during the pandemic, keeping in good health, and personal safety at work.

In Romania, the COVID-19 pandemic brought more sources of stress for nurses, most of them being common with those in other countries: the fear of being infected with COVID-19, social distancing and weaker communication, mask-wearing, shortages in the workforce, too much workload, doctors also affected by stress, and more patients assigned per nurse, with some of them in critical mental conditions. Even if new equipment was received in many hospitals from Romania, many nurses did not have enough qualifications to use it, which created a new source of stress.

Issues related to family put additional pressure on nurses: fear of transmitting the virus to their family; unemployment for some members of the family; online school for children, requiring more supervision from parents; and less time spent with family because of the increasing duties. Moreover, a lack of experience and knowledge to manage an epidemic were important stressors for nurses in this period.

## 5. Conclusions

Job stress is a characteristic of the actual labor market, but the COVID-19 pandemic has enhanced this issue, especially in the case of nurses that are directly implied in the management of this crisis. Knowing the negative effects of stress on normal and physiological hormonal balance, as well as how to cope with stress is vital for nurses to maintain good health and well-being.

The main aim of this paper was the practical evaluation of the vulnerability to stress of nursing students, taking into account their status on the labor market and the period before and during the COVID-19 pandemic. Some demographic variables were analyzed as possible stressors, and females were found to be more vulnerable to stress than males for both categories (employed and non-employed students). Students from a rural environment tended to have higher stress scores than those in urban zones during the pandemic, because of isolation and less opportunities to access the labor market in big cities. Comparisons between nurses who are also students and individuals preparing to work as nurses are also essential to provide recommendations for the latter, based on the experiences of those who worked as nurses during the pandemic. Education remains an important pillar in the fight against stress.

Besides the demographic characteristics analyzed in this paper, there are other important factors that might influence vulnerability to stress and other possible strategies to cope with it. Therefore, this quantitative approach could be completed by qualitative approach-based interviews with nurses to identify other factors that contribute to stress growth and other efficient strategies used in the fight against job stress.

Another limitation of this study was the utilization of a sample of nursing students that is representative only for Bucharest, and not at national level. In future research, data from the rest of the universities in the country with this specialization will be analyzed. However, the empirical findings could serve as recommendations for nursing students and nurses from other developing countries.

## Figures and Tables

**Table 1:** Sample characteristics (overall sample, 526 students).

Variable	Relative Frequency
Gender	21.79% males
	78.21% females
Environment	55.13% in urban environment
	44.87% in rural environment
Age group	18–25 years: 73.08%
	26–29 years: 12.82%
	30–39 years: 3.85%
	40–49 years: 8.97%
	50–65 years: 1.28%
Marital status	83.33% unmarried
	16.67% married
Living conditions	27.56% living alone
	55.13% living with parents
	10.26% living on rent
	7.05% living in student’s house
Status on labor market	64.1% unemployed
	35.9% employed

Source: own calculations in Stata 15, StataCorp LLC, Texas, USA

**Table 2:** The effects of the status on labor market, gender and environment on the stress scores of nursing students before the COVID-19 pandemic using matching methods.

Method	Employed and Non-Employed Nursing Students during the COVID-19 Pandemic				Employed Nursing Students before and during the COVID-19 Pandemic				Females and Males during the COVID-19 Pandemic				Students from Urban Environment and Students from Rural Environment during the COVID-19 Pandemic			
	Coef.	Robust Std. Error	z	sig	Coef.	Robust Std. Error	z	sig	Coef.	Robust Std. Error	z	sig	Coef.	Robust Std. Error	z	sig
Propensity-score matching	5.75	2.39	2.4	0.01	4.46	2.69	1.66	0.09	4.71	2.06	2.28	0.02	4.84	2.24	2.16	0.03
Nearest-neighbour matching	5.87	2.34	2.51	0.01	4.46	2.70	1.65	0.09	4.86	1.90	2.55	0.01	4.81	2.18	2.20	0.02
Regression adjustment	5.38	2.29	2.35	0.01	4.46	2.69	1.67	0.09	4.54	1.92	2.37	0.01	5.28	2.04	2.58	0.01
potential-outcome means	26.79	1.50	17.86	<0	26.17	1.88	13.87	<0	24.03	1.48	16.23	<0	25.14	1.25	19.98	<0

Source: own calculations in Stata 15.

**Table 3:** Differences between females and males before and during the COVID-19 pandemic according to their status in labor market (propensity-score-matching).

Employed Females and Non-Employed Females before the COVID-19 Pandemic				Employed Females and Non-Employed Females during the COVID-19 Pandemic				Employed Males and Non-Employed Males before the COVID-19 Pandemic				Employed Males and Non-Employed Males during the COVID-19 Pandemic			
Coef.	Robust std. Error	z	sig	Coef.	Robust std. Error	z	sig	Coef.	Robust std. Error	z	sig	Coef.	Robust std. Error	z	sig
3.92	2.40	1.63	0.10	2.57	2.99	0.86	0.39	3.98	10.09	0.39	0.69	13.20	1.78	7.40	<0

Source: own computations in Stata 15.

**Table 4:** Comparisons between females and males according to their status in labor market before and during the COVID-19 pandemic.

Method	Non-Employed Females and Non-Employed Males before the COVID-19 Pandemic				Non-Employed Females and Non-Employed Males during the COVID-19 Pandemic				Employed Females and Employed Males before the COVID-19 Pandemic				Employed Females and Employed Males during the COVID-19 Pandemic			
	Coef.	Robust std. d Error	z	sig	Coef.	Robust std. Error	z	sig	Coef.	Robust std. Error	z	sig	Coef.	Robust std. Error	z	sig
Propensity-score matching	1.17	2.93	0.4	0.68	4.71 *	2.06	2.28	0.02	0.09	3.13	0.03	0.97	4.87 *	2.13	2.29	0.02
Nearest-neighbour matching					4.86 *	1.90	2.55	0.01					5.39 *	2.08	2.59	0.01
Regression adjustment					4.54 *	1.92	2.37	0.01					4.90 *	1.98	2.47	0.01
potential-outcome means					24.03 *	1.48	16.23	<0					23.77 *	1.51	15.67	<0

* significant coefficient at 5% level of significance. Source: own computations in Stata 15.

**Table 5:** The impact of various factors on vulnerability to stress during the COVID-19 pandemic.

Source	Partial Sum of Squares	F Calculated	sig
Model	1638.39	2.08	0.03
Environment	415.30	1.59	0.024
Living conditions	395.21	1.68	0.18
Gender	4125.12	5.09	<0
Age group	500.48	1.59	0.18
Marital status	12.33	0.16	0.69

Source: own calculations in Stata 15.

## Data Availability

Data are available by request.
